# Adsorption of tetracycline antibiotic onto modified zeolite: Experimental investigation and modeling

**DOI:** 10.1016/j.mex.2020.100885

**Published:** 2020-04-18

**Authors:** Maryam Dolatabadi, Marjan Mehrabpour, Morteza Esfandyari, Saeid Ahmadzadeh

**Affiliations:** a Student Research Committee, Kerman University of Medical Sciences, Kerman, Iran; b Environmental Science and Technology Research Center, Department of Environmental Health Engineering, Shahid Sadoughi University of Medical Sciences, Yazd, Iran; c Health Sciences Research Center, Department of Environmental Health Engineering, School of Health, Mashhad University of Medical Sciences, Mashhad, Iran; d Department of Chemical Engineering, Faculty of Engineering, University of Bojnord, Bojnord, Iran; e Pharmaceutics Research Center, Institute of Neuropharmacology, Kerman University of Medical Sciences, Kerman, Iran; f Pharmaceutical Sciences and Cosmetic Products Research Center, Kerman University of Medical Sciences, Kerman, Iran

**Keywords:** Artificial Neural Network, Zeolite, Antibiotic, Tetracycline

## Abstract

Artificial Neural Networks (ANNs) model and Adaptive Neuro-Fuzzy Inference System (ANFIS) were used to estimate and predict the removal efficiency of tetracycline (TC) using the adsorption process from aqueous solutions. The obtained results demonstrated that the optimum condition for removal efficiency of TC were 1.5 g L^−1^ modified zeolite (MZ), pH of 8.0, initial TC concentration of 10.0 mg L^−1^, and reaction time of 60 min. Among the different back-propagation algorithms, the Marquardt–Levenberg learning algorithm was selected for ANN Model. The log sigmoid transfer function (log sig) at the hidden layer with ten neurons in the first layer and a linear transfer function were used for prediction of the removal efficiency. Accordingly, a correlation coefficient, mean square error, and absolute error percentage of 0.9331, 0.0017, and 0.56% were obtained for the total dataset, respectively. The results revealed that the ANN has great performance in predicting the removal efficiency of TC.

• ANNs used to estimate and predict tetracycline antibiotic removal using the adsorption process from aqueous solutions.
• The model's predictive performance evaluated by MSE, MAPE, and R^2^.

## Graphical abstract

**Figure:**
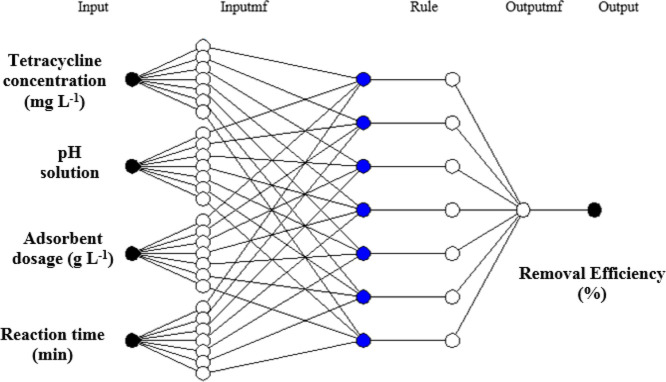

**Table:** Specifications table

Subject Area	Environmental Science
More specific subject area	Wastewater treatment, Modeling, Artificial Neural Networks (ANNs) model
Method name	Artificial Neural Networks (ANNs) model and Adaptive Neuro-Fuzzy Inference System (ANFIS), Modified adsorbent preparation method
Name and reference of original method	M. Dolatabadi, M. Mehrabpour, M. Esfandyari, H. Alidadi, M. Davoudi, Modeling of simultaneous adsorption of dye and metal ion by sawdust from aqueous solution using of ANN and ANFIS, Chemometrics and Intelligent Laboratory Systems 181 (2018) 72–78.
Resource availability	

## Method details

### Adsorption experiments and instrumental analyses

Batch experiments were conducted in a plexiglass reactor that contained a 100 mL sample of specified TC concentration at room temperature (22 ± 2 °C). After pH adjustment at the desired level, the required amount of modified zeolite (MZ) was added to the reactor and blended at 100 rpm. At the end of the given reaction time, the suspension separated by was centrifuged at 3000 rpm for 5 min and the supernatant analyzed for residual TC. The fortified distilled water (distilled water containing TC) was prepared by adding a certain amount of TC into the distilled water, whereas the real wastewater was obtained by sampling the hospital wastewater.

The concentration of TC after each run measured by KNAUER HPLC using a C_18_ column (150 mm, 4.6 mm, 5 µm). The mobile phase consists of 75% oxalic acid (10 mM), 13% of methanol, and 12% acetonitrile with a flow rate of 1 mL min^−1^. The temperature of the oven and the wavelength of the UV detector set to 30 °C and 360 nm, respectively [1].

The removal efficiency of TC through the adsorption process was calculated by Eq. (1) [2]:

(1) $$\text{Removal}\;\left(\%\right)=\;\frac{C_{0}-C_{t}}{C_{0}}\times \;100$$

where *C*_0_ and *C_t_* (mg L^–1^) denote the TC concentration before adsorption and at the time *t*, respectively.

### Characteristics of employed real wastewater

The characteristics of the employed real wastewater included the total suspended solids (TSS) of 121±14 mg L^−1^, total dissolved solids (TDS) of 627±34 mg L^−1^, temperature of 21±2 °C, chemical oxygen demand (COD) of 734±42 mg L^−^ ^1^, biochemical oxygen demand (BOD5) of 314±27 mg L^−1^, pH of 6.4 ± 0.7, and electrical conductivity (EC) of 1172±49 µmho cm^−1^.

### Description of ANN method

**Fig. 1:**
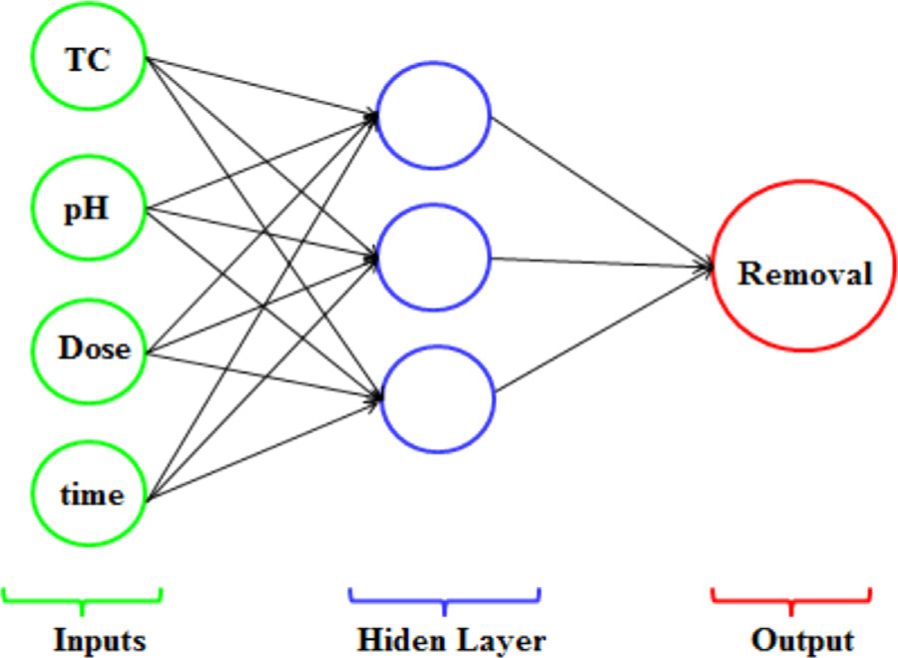
Schematic of the ANN model used in the current study.

The data was prepared to enter into the ANN model. The Matlab (version 2014b) was used for computer coding all the calculations required. The best model predicted including the best learning and experimental set, normalizing the data, the number of hidden layers and neurons per layer, the functions of transfer, conversion, and operation, as well as the learning rate, the number of repetitions and the algorithm of teaching. In this study, the data set selected randomly for train and validation was 74%, 26%, respectively. The data analysis was performed by Microsoft office 2010 software. There are no systematic methods in determining the mentioned cases, so the best design of the network is achieved through experience and Trial and error. The schematic of the ANN model used in the current study presented in Fig. 1.

The training flowchart of ANN network has three layers: an input layer, an output layer and an hidden layer. The inputs to a neuron comprise its bias and the sum of its weighted input. The output value of a neuron depends on the neuron's inputs as well as transfer function [3]. In mathematical terms, a neuron can be described as follows:

(2) $$u_{k}=\sum_{j=1}^{m}w_{kj}x_{j}$$

(3) $$y_{k}=\varphi \left(u_{k}+b_{k}\right)$$

where $x_{1},\ldots ,x_{m}$ are the inputs;$w_{k1},\ldots ,w_{km}$ are the weights of neuron; *u_k_* is the linear combiner output due to the input signals; φ is the activation function(for this paper used Log-sigmoid);*b_k_* is the bias or threshold; and *y_k_* is the output signal of the neuron.

### Description of ANFIS method

**Fig. 2:**
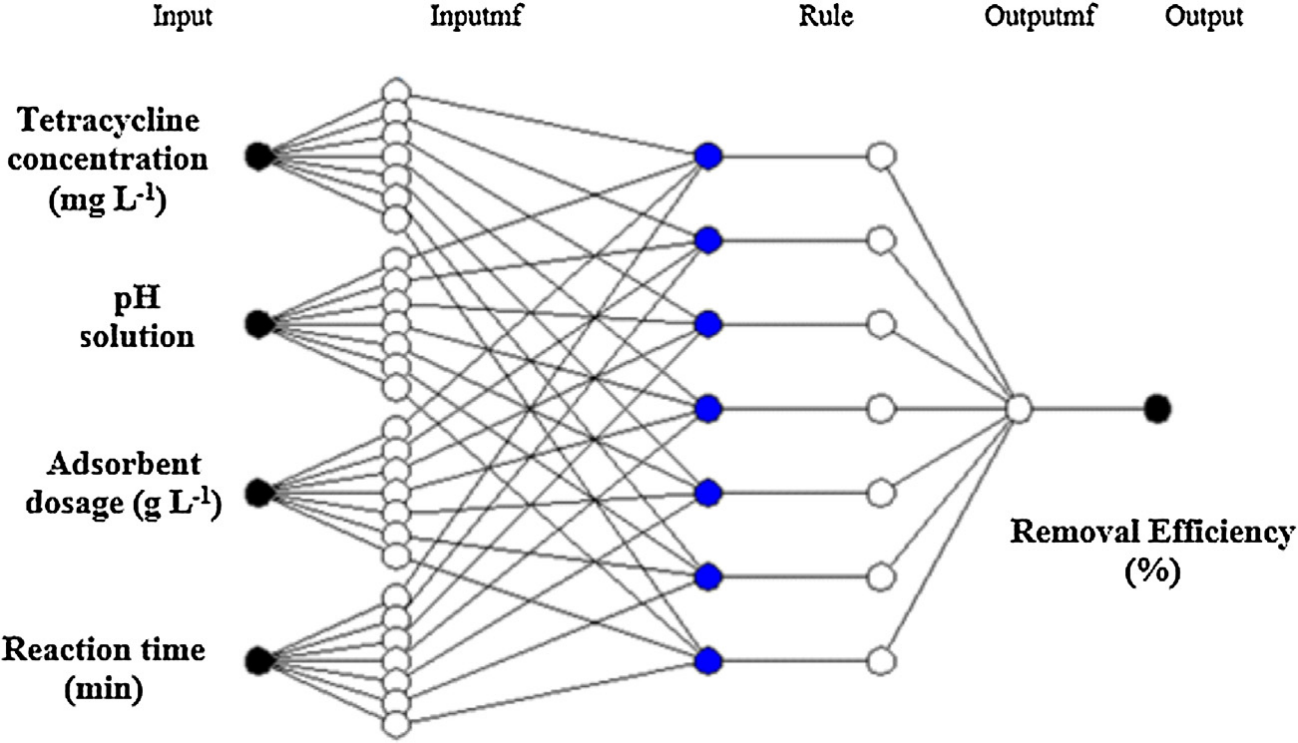
The architecture of the ANFIS model for predicting the removal efficiency of TC.

**Fig. 3:**
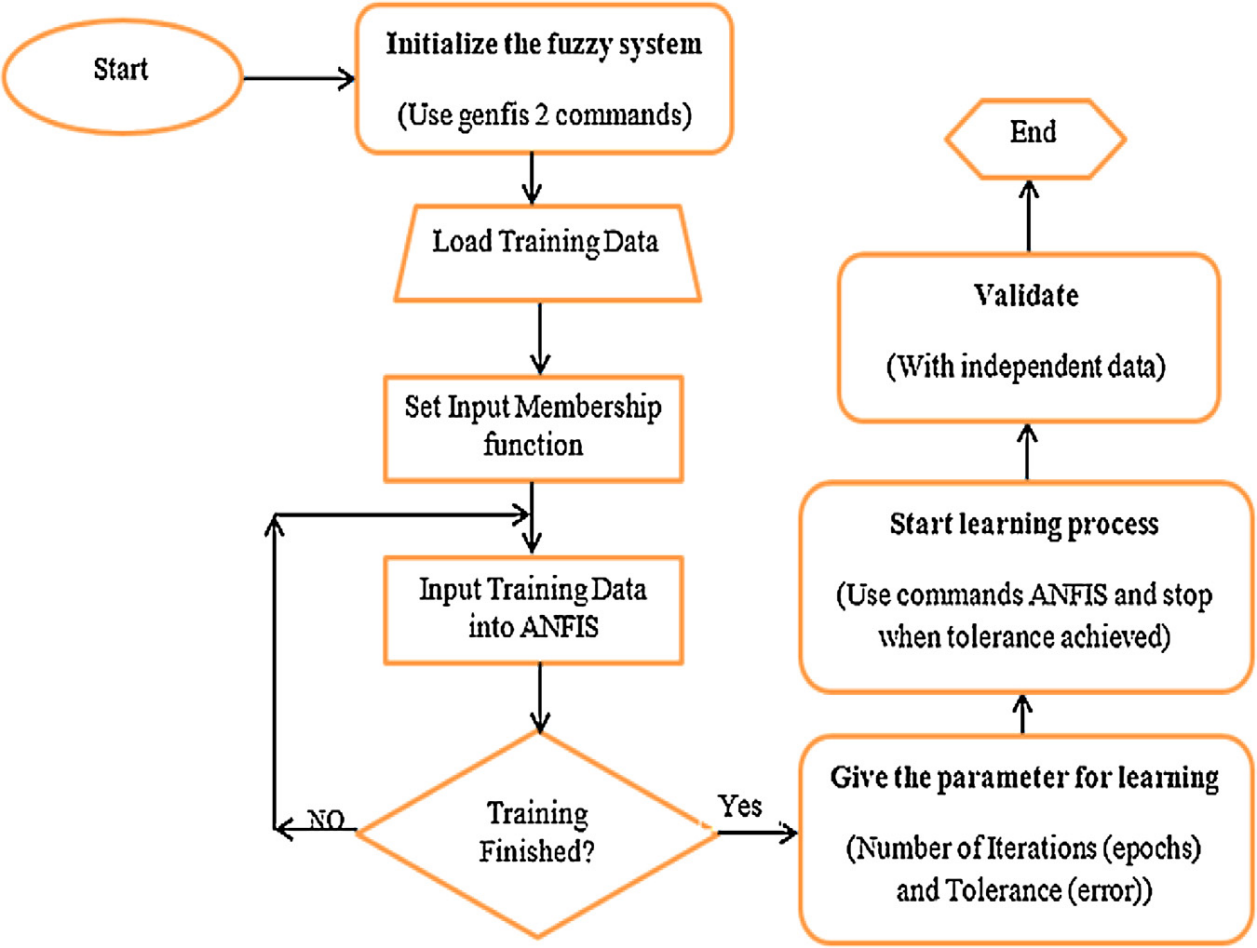
Flowchart of ANFIS modeling for removal of TC in the current study.

Adaptive Neuro-Fuzzy Inference System (ANFIS) combines the learning capacities of artificial neural networks (ANNs) and reasoning capacities of fuzzy systems [4]. The learning algorithm for ANFIS is a hybrid algorithm, which is a combination of gradient descent and the least-squares method [5]. The consequent parameters were optimized under the condition that the premise parameters remain fixed. The main benefit of the hybrid approach is that it converges much faster since it reduces the search space dimensions of the original pure back-propagation method used in neural networks. The ANFIS structure and research procedure are presented in Figs. 2 and 3, respectively [6,7].

### Statistical modeling

To achieve acceptable results through the neural network, the selection of appropriate data as inputs and outputs of the network is required. In the current work, 30 experimental data were used for modeling. Different modes were investigated to determine the best model used. Where, the best model was selected using the highest correlation coefficient. In the current work, a total of 30 run experiments were used for the models. To determine the best input pattern for the network, the effective variables on the removal efficiency were data processed, calculated and matrixed. The model included an input layer with four neurons (TC initial concentration, pH, adsorbent dosage, and reaction time), a hidden layer with six different states, and an output layer with a neuron (removal efficiency of TC). Before starting the learning process, to avoid some educational damages and to ensure that the network not be saturated with large numerical amounts of weight, the normalization of the input data in the range from zero to one calculated completely randomized by Eq. (4).

(4) $$X_{\text{norm}}=\frac{\left(X-X_{\text{min}}\right)}{\left(X_{\text{max}}-X_{\text{min}}\right)}$$

where *X*_norm_ denotes the value of the normalized data and *X, X*_max_ and *X*_min_ denote the amount of input, maximum and minimum data, respectively. Finally, for comparison, the normalized data returned to the original *X* data after modeling according to Eq. (5).

(5) $$X=\left[X_{\text{norm}}\left(X_{\text{max}}-X_{\text{min}}\right)\right]+X_{\text{min}}$$

At the learning phase of the network, there are various criteria for stopping the neural networks. These criteria include the computational time, the error rate, and the number of steps to apply the data to the network. Relationships such as the mean correlation coefficient (R^2^), mean square error (MSE), and mean absolute percentage error (MAPE) were used to calculate the error rate. The correlation coefficient was expressed by Eq. (6) as follows:

(6) $$R^{2}=\frac{{\left(\sum_{i=1}^{n}\left(y_{di}-{\bar{y}}_{di}\right)\left(y_{i}-{\bar{y}}_{i}\right)\right)}^{2}}{\sum_{i=1}^{n}{\left(y_{di}-{\bar{y}}_{di}\right)}^{2}{\left(y_{i}-{\bar{y}}_{i}\right)}^{2}}$$

where *y_i_, y_di_*, and n denote the predicted data, the real data, and the total number of data, respectively.

To evaluate the prediction performance of the developed model, the statistical standards including mean absolute error (MAE), mean squared error (MSE), root mean squared error (RMSE) and mean absolute percentage error (MAPE) were defined as below:

(7) $$\text{Mean}\;\text{absolute}\;\text{error(MAE)}\;=\frac{\sum_{i=1}^{n}\left|{y^{\prime }}_{i}-y_{i}\right|}{n}$$

(8) $$\text{Root}\;\text{mean}\;\text{squared}\;\text{error}\;\text{(RMSE)}\;=\sqrt{\frac{\sum_{i=1}^{n}{\left({y^{\prime }}_{i}-y_{i}\right)}^{2}}{n}}$$

(9) $$\text{Mean}\;\text{absolute}\;\text{percentage}\;\text{error}\;\text{(MAPE)}\;=\frac{1}{n}\sum_{i=1}^{n}\left|\frac{{y^{\prime }}_{i}-y_{i}}{y_{i}}\right|$$

Where *y_i_* is the actual value and $y_{i}^{\prime }$ is the predicted value for the train and test.

### Acquired data from developed ANN model

The most important factors in the modeling of the neural networks which have a direct effect on the model output are the main variables (the number of hidden layers and number of neurons). Therefore, the modeling process was initiated from a hidden layer, in which a neuron with six different states were selected. As seen from Table 1, the correlation coefficient for training, validation, and total data was calculated for each state and the highest correlation coefficient were used (10–10).

**Table 1:** The best *R*^2^ value for ANN with a different structure.

No	Hidden layer	*R*^2^		
		Train	Validation	Total
1	[5]	0.981	0.621	0.906
2	[10]	0.991	0.788	0.882
3	[15]	0.991	0.858	0.926
4	[5–5]	0.839	0.797	0.799

**Fig. 4:**
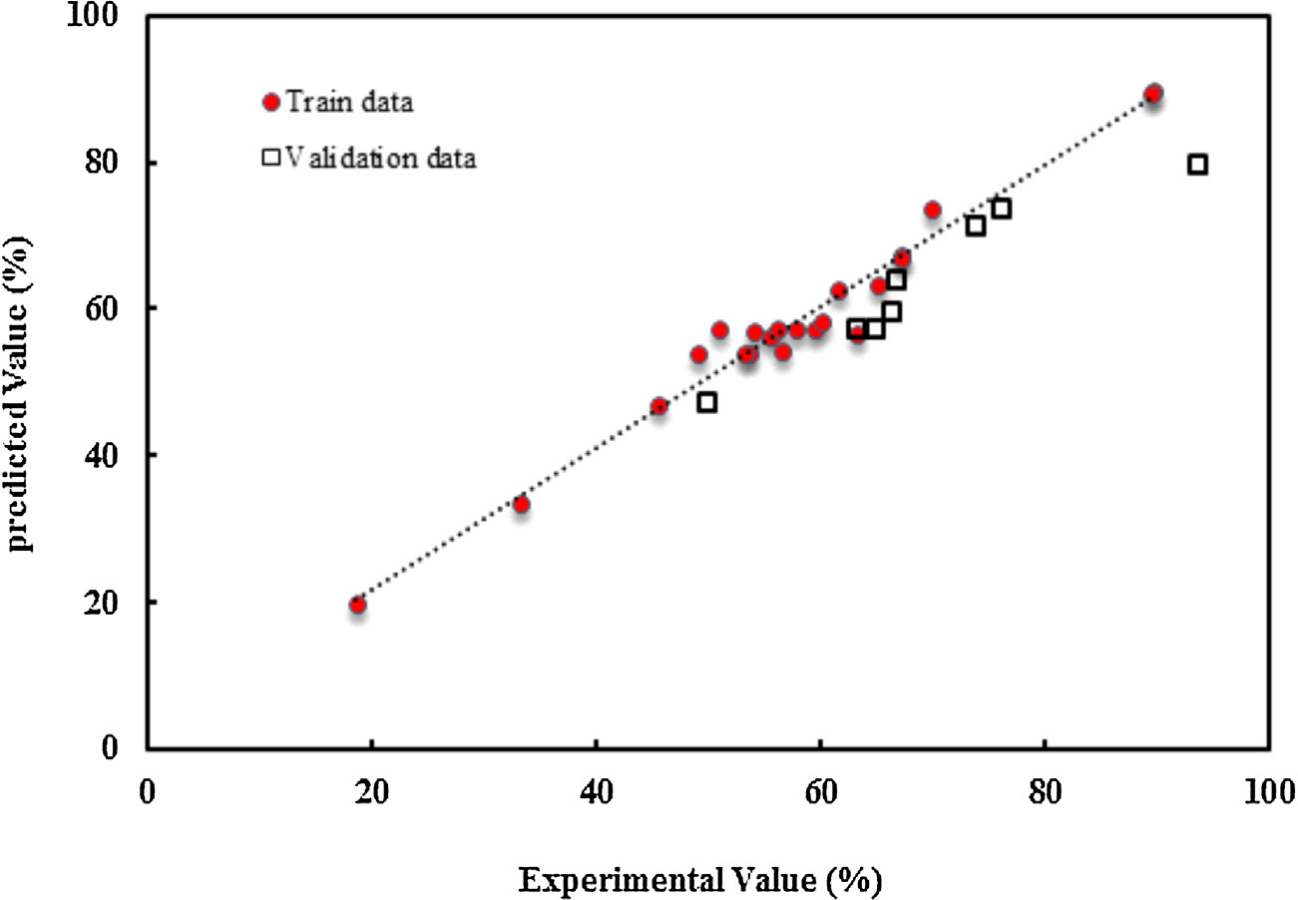
Correlation between experimental and predicted values of TC removal efficiency using the ANN model for train and validation data.

After performing the training and validating phases of the neural networks, the algorithm was designed to be able to examine all possible scenarios for the number of layers, the number of neurons per layer, different transfer functions, and also different training algorithms. 74% and 26% of input data were used for network training and validation data, respectively, and a lower coefficient was applied to the error value derived from training data so that the results are less affected by this parameter. Validation and training samples must be different and are selected randomly from the original data set, which used the “randperm” function in Matlab. The Levenberg–Marquardt algorithm with the least error in training and the goal value of 10–5 was selected as the best backpropagation algorithm. The configuration parameters of this network were obtained by the trial and error method. The performances of these networks were evaluated by error and regression parameters and correlation coefficient. The network with the least deviation from the target values for both training data and network validation data was offered as a model. The modeling data against the experimental data was depicted in Fig. 4.

As seen from the comparison of simulated values with real values, the artificial neural network models were presented in the current study as advanced tools with high potential could well predict the removal efficiency of TC.

### Acquired data from developed ANFIS model

Two models of Sugeno, with automatic extraction of data from AFIS [GENFIS2], were used. The MATLAB software was adopted for comparison purposes. Moreover, the coverage threshold fixed to 0.01. Table 2 shows the used ANFIS information in the current study with the Back-propagation optimum method for the removal efficiency of TC.

**Table 2:** The ANFIS information used in the current study.

Characterization	Value
Number epoch's	1000
Number of nodes	257
Number of linear parameters	125
Number of nonlinear parameters	200
Total number of parameters	30
Number of fuzzy rules	25

**Fig. 5:**
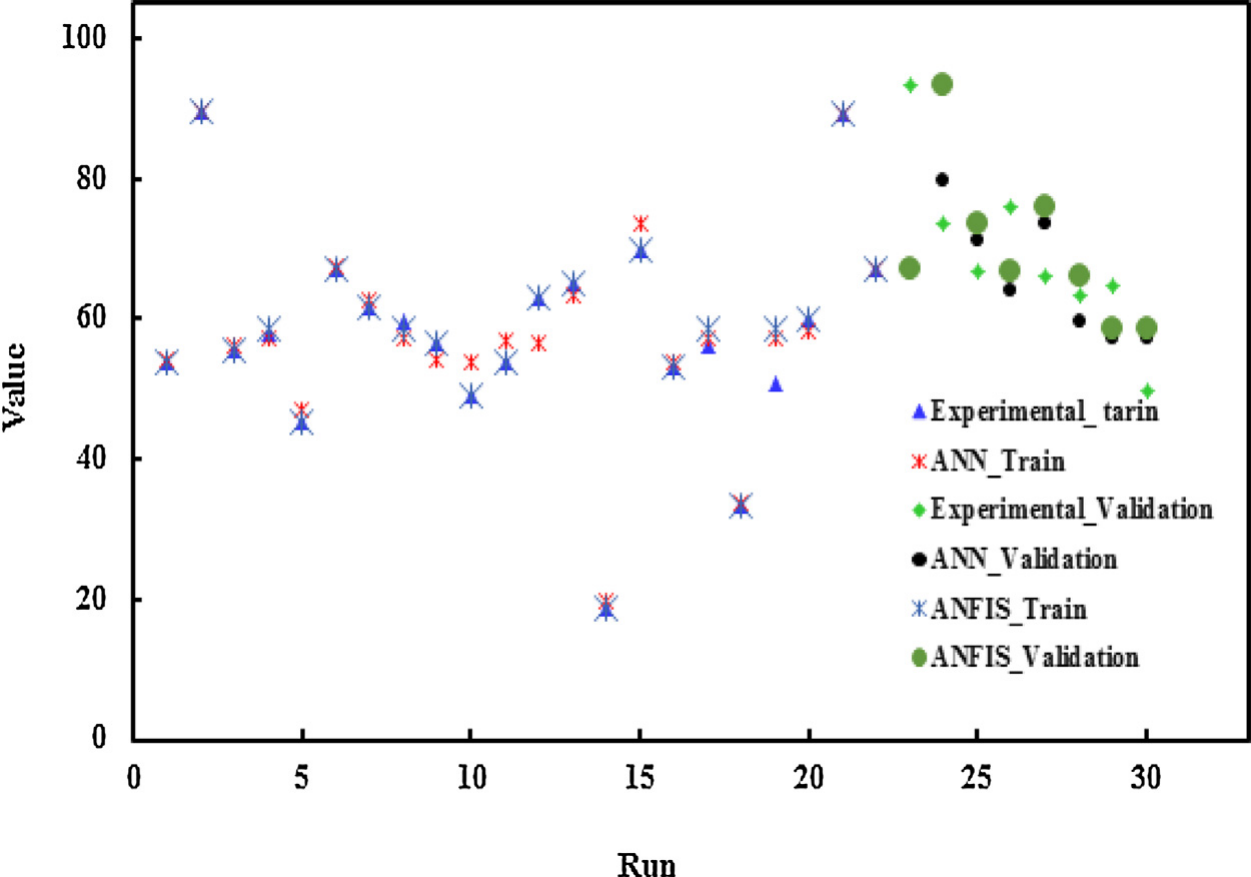
Comparison of the experimental and predicted results for removal efficiency of TC in ANN and ANFIS models.

In Figs. 4 and 5, the desired values (removal efficiency of TC) versus ANFIS predictions for the verification data points were demonstrated. These figures reveal that an acceptable agreement between the predicted and experimental data achieved.

### Comparison of ANN and ANFIS models

For the ANN model, the low values of the conventional error functions: MAE=2.832, RMSE=4.117, and MAPE=0.046 are in an acceptable range. Besides, the correlation coefficients are greater than 0.9331, which indicated a satisfactory adjustment between the experimental data and those predicted by ANN model. While for the ANFIS model, low values of MAE=0.759, RMSE=2.065, and MAPE=0.013, are in agreement with the reported range in the literature [8], [9], [10], [11]. Furthermore, the correlation coefficient than found by ANN is closer to 1.00, which implies excellent agreement between the experimental data and those predicted by the ANN model. Table 3 listed the values of MAE, RMSE, MAPE and correlation coefficient (R^2^) of ANN and ANFIS models for prediction of TC removal efficiency.

**Table 3:** MAE, RMSE, MAPE and Correlation coefficient (*R*^2^) for removal efficiency of TC by ANN and ANFIS models.

Model	ANN model				ANFIS model			
Type of Error	MAE	RMSE	MAPE	Correlation coefficient (*R*^2^)	MAE	RMSE	MAPE	Correlation coefficient (*R*^2^)
Train	1.854	2.652	0.035	0.9695	0.552	1.787	0.01	0.9867
Validation	5.524	6.649	0.077	0.9144	1.328	2.686	0.021	0.9674
Total	2.832	4.117	0.046	0.9331	0.759	2.065	0.013	0.9811

The predicted removal efficiency of TC by ANN and ANFIS models were compared with the experimental results in Fig. 5.

### Treatment of real wastewater and validity of the model

The removal efficiency of the treatment process through the adsorption of TC onto the modified zeolite was investigated for both fortified distilled water and real wastewater samples in five various operating conditions with superior desirability of 99%. All experiments were performed in triplicate. As seen in Table 4, a satisfactory agreement was observed between the TC removal efficiency in fortified distilled water and real samples, however, a slight decrease in the efficiency was observed in real samples due to their complex matrix. Desirability is one of the most frequently used response optimization parameters employed for the analysis of experiments to evaluates the extent of access to the optimal conditions and ranging from 0 to 1 (least to most desirable, respectively) [12,13]. To appraise the reliability of optimum conditions predicted by the empirical model, a set of experiments was carried out testing the model. It is found that the deviation is smaller between the fortified distilled water sample and real sample, and this result further confirms the validity of the model.

**Table 4:** Comparison of TC removal efficiency for both fortified distilled water and real wastewater samples.

No.	Initial TC concentration (mg L^−1^)	pH	Adsorbent Dosage (g L^−1^)	Reaction time (min)	Removal efficiency (%)		Desirability
					fortified distilled water sample	Real sample	
I	10	8.0	1.5	60	95.23	93.82	0.999
II	10	7.5	2.0	60	93.39	90.71	0.998
III	5	7	1	30	84.48	81.37	0.997
IV	20	4	1.5	45	44.18	41.65	0.996
V	15	10	1.5	45	37.80	36.42	0.998

## Conclusion

In the current work, the neural network models successfully were developed to simulate the removal efficiency of TC in aqueous solution. The best network structure based on the lowest error found to be the Levenberg–Marquardt algorithm, which included four inputs, one output and ten neurons in the first layer. In the training phase, the neural network learns the relationship between the variables with the help of input data. In the validation phase, the prediction of the data values calculated as output. Consequently, the predicted results were compared with the actual values and the error rate was calculated. The error values should be at their lowest levels, for which purpose the network first designed and the training and validation practice were repeated several times to minimize the error. To evaluate and compare the results provided by the methods and error estimation, the mean square error (MSE) and mean absolute percentage error (MAPE) were calculated. Since the accuracy of the obtained results from the neural networks directly related to the accuracy and range of numbers used in the validation and network training phases, therefore, the reliable and high-precision data were used in the mentioned phases. Moreover, due to the high speed of computing in neural networks, this model was employed as an efficient model for predicting data in environmental sciences.

## Declaration of Competing Interest

The authors declare that they have no known competing for financial interests or personal relationships that could have appeared to influence the work reported in this paper.

## Appendix Supplementary materials

Image, application 1
