# Supplementary material for: Adsorption of tetracycline antibiotic onto modified zeolite: Experimental investigation and modeling
Source: MethodsX. 2020 Apr 18;7:100885. doi: 10.1016/j.mex.2020.100885 (PMC7184631; doi:10.1016/j.mex.2020.100885)
Supplement: Supplementary file 1 [file mmc1.docx]

**Manuscript ID:** MEX-D-20-00017

**Title:** Adsorption of tetracycline antibiotic onto modified zeolite: experimental investigation and modeling.

Additional information

**Preparation of modified zeolite**

Clinoptilolite zeolite as natural zeolite purchased from Semnan Negin Powder Co. in Iran. Its particle size was 60-70µm. For zeolite modifying, first, it was washed several times with dilution water, then it was exposed to the temperature at 250 °C for 12 h (in order to organic matter destruction). Then, it was shaking for 48 h in a 1M of NaCl solution in temperature at 25 °C. Zeolite washed by deionized water. Finally, after zeolite surface preparation it's modifying was begun by the Hexadecyltrimethylammonium bromide surfactant. For this purpose, 0.01 M solution of surfactant in the 1:20 (V/W) ratio (surfactant: zeolite) prepared. The solution was shaken for 6 h by the magnetic stirrer and then modified zeolite particles were dried at about 12 h in the oven at 25° C [1]. The obtained modified adsorbent in this method was named MZ.

**Characterization of adsorbent (MZ)**

The Fourier transforms infrared (FTIR) spectra (400–4000 cm^-1^) of MZ as adsorbent before and after TC adsorption were recorded by a Thermo-Nicolet AVATAR 370 FT-IR Spectrometer.

Figure 1s revealed the FTIR spectra of the clinoptilolite zeolite as natural zeolite (NZ) and the modified zeolite (MZ). The zone at 3150-3600 cm^-1^ attributed to the hydroxyl stretching region of NZ water, its intensity decreases with elevating temperature because of the dehydrate in MZ. The sharp peak at 1047 cm^-1^ is assigned to the bending vibration band of Si(Al)-O in tetrahedral Si(Al)O_4_ in NZ. The structural bands at 450-900 cm^-1^ are responsible for the bending vibrations of T-O, T-O-T, and O-T-O bonds in tetrahedral SiO_4_ and AlO_4_ [2]. Compared to the spectrum of NZ, the MZ exhibits three additional absorption bands at The 1430 and 1630 cm^-1^ peaks relation to C=C skeletal vibration, and the 3020 cm^-1^ corresponded to the C–H stretching, which may be a proof of the combination of NZ and surfactant.

**Figure 1s. FT-IR spectra of NZ and MZ.**

Microscopic characterization performed using a Zeiss (LEO) 1450VP Scanning Electron Microscope (SEM), to observe the surface microstructure of the prepared adsorbent. The SEM image of MZ (Figure 2s) revealed that MZ surface characterized by a porous and coarse texture, therefore suggesting uneven and numerous adsorption of TC onto the whole structure.

**Figure 2s. SEM micrograph of MZ.**

The point of zero charges (pH_pzc_), that implies the neutral surface charge of MZ, was determined for MZ using metrohm pH meter (model 827 pH lab) apparatus. The pH_pzc_ of MZ specified as 7.6. It implies that the surface of MZ would be positively charged at pH below 7.6 and negatively charged at solution pH more than 7.6, while it is neutral at pH 7.6.

**Real sample preparation**

The experimental extraction procedure of TC by dispersive solid-phase microextraction (dispersive-SPME) method carried out in seven step as follow [3]:

(1) 5 mL of MeCN and 50 µL of 70- 2% perchloric acid are added to 4 mL of water in a 50 mL centrifuge tube and shake the mixture vigorously for 1 min by using Vortex mixer at maximum speed.

(2) 2 g anhydrous magnesium sulfate and 1 g sodium chloride, mix on a Vortex mixer immediately for 1 min and then centrifuge for 4 min at 4000 rpm.

(3) Transfer 1.5 mL aliquot of upper MeCN layer into 2 mL minicentrifuge vial containing 30 mg dispersive PSA sorbents, mix and held for 10 min. (primary and secondary amine (PSA))

(4) Add 10 µL of 1 M NaOH solution, mix immediately on a Vortex mixer for 10 s, and centrifuge for 1min at 6500 rpm.

(5) Remove the solvent with a glass pipette as clean as possible.

(6) Dry the solid sorbents under a nitrogen stream and stir these sorbents manually with a small glass rod to accelerate drying times if necessary.

(7) Add 300 µL of desorption solution (10:2:88 (v/v/v) MeCN/ 70–72% perchloric acid/ water) to the dried sorbents, mix on Vortex mixer for 30 s, and centrifuge for 1min at 6500 rpm. An aliquot (50 µL) injected into the HPLC. For real wastewater samples, repeated two cycles of adsorption steps 3–5 required for the adequate sensitivity of TC by using HPLC. All samples analyzed soon after preparation.

**Effect of Independent Variables on TC Removal onto MZ**

Figure 3s demonstrated the effect of initial TC concentration in the range of 5 to 30 mg L-1 on the removal efficiency of TC using MZ. As seen, the removal efficiency decreased from 84.4 % to 37.8 % with an addition of TC concentration from 5 mg L-1 to 30, respectively. Previous studies revealed a general trend that indicated that by increasing the initial target concentration the removal efficiency of the process decreased. many authors have ascribed this to the saturation of adsorption sites on the sorbent surface by the contaminant of species [4, 5].

**Figure 3s. Effect of Initial TC concentration on the removal efficiency of TC (MZ dosage=10 mg L^-1^, and pH=7).**

Moreover, the effect of reaction time on the adsorption of TC by MZ evaluated (Figure 3s). The experiments showed that the adsorption increased quickly up to 75 % in the first 45 min, and there after it decreased until attaining adsorption equilibrium at 60 min. The reaction time axis showed that the process of adsorption was initially rapid which later slowed and eventually attained equilibrium at 60 min [6, 7]. The adsorbent dosage is a key factor that influences the adsorption process of the adsorbate because the adsorption is highly dependent upon the accessibility of adsorption sites of the adsorbent. The effect of adsorbent dosage on the removal efficiency of TC examined, and the obtained results demonstrated in Figure 4s. As seen, by increasing the MZ adsorbent dosage from 0.5 to 2 g L-1, the TC removal efficiency gradually increased from 59.5 to 94.6 %. The increase in the adsorption efficiency attributed to the presence of a higher number of adsorption active sites at a higher adsorbent dosage. Nonetheless, a further rise in the adsorbent dosage from 0.5 to 1.5 g L-1 resulted in a constant TC removal efficiency (see Figure 4s). The observed behavior is because of the amount of adsorption active sites on the adsorbent surface is much bigger than the amount of adsorbate in solution when adsorbent dosage reached a certain value, resulting in no more increase in the adsorbate removal efficiency.

**Figure 4s. Effect of MZ dosage on the removal efficiency of TC (Initial TC concentration=10 mg L^-1^, and pH=7).**

The pH recognized as a very important factor that rules the adsorption process. It confirmed that the pH affects the surface charge of the adsorbent as well as the speciation of adsorbate and the degree of ionization. The effect of solution pH on the removal efficiency of TC presented in Figure 5s. The removal efficiency of TC was low under acidic conditions and increased with increasing the pH to 8. further increase in the pH to 11 resulted in decreased inTC removal efficiency. TC as an amphoteric compound with several functional groups such as amino, phenol and, alcohol has three pK_a_ values (3.30, 7.68 and 9.68), thus it may predominately exists as a cation (+00) (below pH 3.30), due to the protonation of dimethyl-ammonium group, and exists as a zwitterion (±0) (between pH 3.30 and 7.68), when the proton of phenolic diketone moiety is lost and exists as an anion (+ – or 0-) (above pH 7.68) due to the loss of protons from the phenolic diketone moiety and tricarbonyl system [8, 9]. This behavior attributed to the pH_pzc_ of MZ, which found to be 8.3. At pH˂8.3, the MZ surface was positively charged, and the TC sorption enhanced by the electrostatic attraction between them, whereas, at pH˃8.3, the MZ surface was negatively charged, thereby affecting TC adsorption capacity due to the anionic nature of both sorbent and sorbate. That being so, the maximum removal efficiency of TC occurred at pH 8.0.

**Figure 5s. Effect of pH on the removal efficiency of TC (Initial TC concentration=10 mg L^-1^, MZ dosage=1.5 g L^-1^, and reaction time=60 min).**

**References:**

[1] N. Liu, M.-x. Wang, M.-m. Liu, F. Liu, L. Weng, L.K. Koopal, W.-f.J.J.o.h.m. Tan, Sorption of tetracycline on organo-montmorillonites, 225 (2012) 28-35.

[2] M. Huang, C. Xu, Z. Wu, Y. Huang, J. Lin, J. Wu, Photocatalytic discolorization of methyl orange solution by Pt modified TiO2 loaded on natural zeolite, Dyes and Pigments 77(2) (2008) 327-334.

[3] W.-H. Tsai, T.-C. Huang, J.-J. Huang, Y.-H. Hsue, H.-Y.J.J.o.C.A. Chuang, Dispersive solid-phase microextraction method for sample extraction in the analysis of four tetracyclines in water and milk samples by high-performance liquid chromatography with diode-array detection, 1216(12) (2009) 2263-2269.

[4] M.T. Yagub, T.K. Sen, S. Afroze, H.M. Ang, Dye and its removal from aqueous solution by adsorption: a review, Advances in colloid and interface science 209 (2014) 172-184.

[5] T. Ngulube, J.R. Gumbo, V. Masindi, A. Maity, An update on synthetic dyes adsorption onto clay based minerals: a state-of-art review, Journal of environmental management 191 (2017) 35-57.

[6] W. Liu, X. Shen, Y. Han, Z. Liu, W. Dai, A. Dutta, A. Kumar, J. Liu, Selective adsorption and removal of drug contaminants by using an extremely stable Cu (II)-based 3D metal-organic framework, Chemosphere 215 (2019) 524-531.

[7] A. Abd-Elhamid, E.A. Kamoun, A.A. El-Shanshory, H.M. Soliman, H. Aly, Evaluation of graphene oxide-activated carbon as effective composite adsorbent toward the removal of cationic dyes: Composite preparation, characterization and adsorption parameters, Journal of Molecular Liquids 279 (2019) 530-539.

[8] G. Li, D. Zhang, M. Wang, J. Huang, L. Huang, Preparation of activated carbons from Iris tectorum employing ferric nitrate as dopant for removal of tetracycline from aqueous solutions, Ecotoxicology and environmental safety 98 (2013) 273-282.

[9] Y.A. Ouaissa, M. Chabani, A. Amrane, A. Bensmaili, Removal of tetracycline by electrocoagulation: kinetic and isotherm modeling through adsorption, Journal of Environmental Chemical Engineering 2(1) (2014) 177-184.
